# Improving the robustness of analog deep neural networks through a Bayes-optimized noise injection approach

**DOI:** 10.1038/s44172-023-00074-3

**Published:** 2023-05-11

**Authors:** Nanyang Ye, Linfeng Cao, Liujia Yang, Ziqing Zhang, Zhicheng Fang, Qinying Gu, Guang-Zhong Yang

**Affiliations:** 1 Shanghai Jiao Tong University, Shanghai, P.R. China; 2 University of Cambridge, Cambridge, United Kingdom; 3 Shanghai Artificial Intelligence Laboratory, Shanghai, P.R. China

**Keywords:** Electrical and electronic engineering, Computer science

## Abstract

Analog deep neural networks (DNNs) provide a promising solution, especially for deployment on resource-limited platforms, for example in mobile settings. However, the practicability of analog DNNs has been limited by their instability due to multi-factor reasons from manufacturing, thermal noise, etc. Here, we present a theoretically guaranteed noise injection approach to improve the robustness of analog DNNs without any hardware modifications or sacrifice of accuracy, which proves that within a certain range of parameter perturbations, the prediction results would not change. Experimental results demonstrate that our algorithmic framework can outperform state-of-the-art methods on tasks including image classification, object detection, and large-scale point cloud object detection in autonomous driving by a factor of 10 to 100. Together, our results may serve as a way to ensure the robustness of analog deep neural network systems, especially for safety-critical applications.

## Abstract (web summary)

Device imperfections are a major challenge that limit the potential of analogue neural networks. Nanyang Ye and colleagues propose a training-time noise injection approach to improve their robustness without hardware modifications, which comes with theoretical guarantees.

## Introduction

Recently, analog DNNs have emerged as a promising direction to further alleviate the speed power consumption limits of standard von Neumann computational architectures. For example, with the crossbar computing architecture, a common operation in DNNs—in-place dot product, can be efficiently implemented by analog computation without the need to transfer data from memories to computing devices, therefore breaking the memory wall, which limits the efficiency of existing deep-learning accelerators. This is in contrast to standard von Neumann architectures, where data has to be moved to computation units before computation.

However, challenges exist in that analog DNNs are not compatible with current deep-learning paradigms designed primarily for deterministic circuits. Without the potential gap between high and low voltages to resist noise, as in digital circuits, the stability of analog DNNs is rather sensitive to thermal noise, electrical noise, process variations, and programming errors. As a result, the parameters of DNNs represented by the conductance at each crossbar intersection can be easily distorted, jeopardizing the utility of analog deep-learning systems, especially for life-critical applications^1^.

Many efforts have been made to minimize the detrimental effect of noise by improving the device stability from the engineering perspective, such as employing novel materials and optimizing the design of circuits^2–7^. These approaches can mitigate the issue to some extent, for example, in a certain field or for single tasks. However, hardware modification is normally on a specific type or types of devices that lacks universality in manufacturing, and will bring extra hardware costs. From an algorithmic perspective, previous work has shown that noise injection during training could lead to empirical improvements in the noise resilience of analog computing devices. For example, standard Gaussian noise is widely introduced in the training process of DNNs to improve the robustness^8–10^, while the improvement depends on the measurements of the time-consuming noise for each single device to be deployed. Also, prior studies focused more on the methods themselves but lacked in-depth analysis such as explanations about how to choose the strength of the injected noise and how the noise spectrum would affect the performance^8–13^. Therefore, the fundamental understanding is still unclear which limits the wide application of analog DNNs in real-world situations. Consequently, developing a theoretically guaranteed method to ensure the robustness of analog DNNs could be essential for their widespread utility and may lead to improvements in life-critical applications, such as autonomous driving.

Herein, we present a thorough theoretical study and a theoretically guaranteed noise injection approach that allows us to train an analog DNN that is fault-tolerant, robust against noise, and generalizable to complex tasks. Inspired by previous neuroscience research that demonstrated the benefits of noise in human neural systems^14^, we demonstrate a noise injection approach leveraging the Bayesian optimization method—“BayesFT”—to optimize the characteristics of the injected noise, therefore enhancing the robustness of analog neural networks. Compared with some typical state-of-the-art studies (Supplementary Table 1), we delivered more comprehensive studies considering many types of injected noise with theoretical proofs to obtain robust analog DNNs. Further performance evaluation experiments prove the effectiveness, generalizability and practicality of our method.

## Results

### Discussions on key factors affecting fault tolerance

**Fig. 1:**
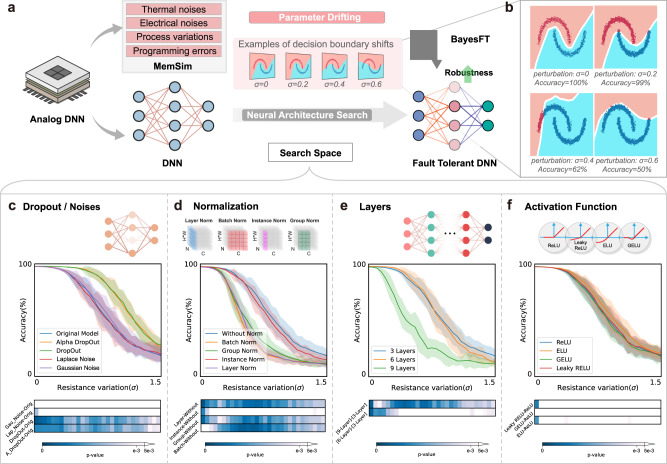
The framework of fault-tolerant analog deep neural networks. **a** The accuracy of analog DNNs is limited by noise and variations of analog devices. A two-class classification problem, as an example, is shown that upon parameter drifting, the decision boundary shifts. Bayesian optimization (BayesFT in the figure) is employed to improve the robustness of analog DNNs by finding the optimal neural network settings. **b** A zoom-in plot of “Examples of decision boundary shifts” in (**a**). Pink and blue shadings indicate the two classes, and the dispersed dots are the decisions. The decision dots should be in the shading area with the same color ideally (i.e., accuracy = 100%). Analog noise perturbs DNNs and shift the boundary to a lower accuracy. **c** Adding inductive noise can improve the robustness of analog DNNs. Alpha dropout, dropout, Laplace noise, and Gaussian noise were examined, and the first two showed improved accuracy compared to the original model at different resistance variations (*σ*). **d** Normalization has negative effects on analog DNNs' robustness. Batch normalization, layer normalization, instance normalization, and group normalization were examined and showed worse performance. **e** Increasing the model complexity leads to degenerated robustness. The original model with three layers can achieve higher accuracy compared to the six-layer and nine-layer models. **f** Nonlinear activation functions have negligible effects on the robustness of DNNs. The performance of the original model with the ReLU function and models with nonlinear activation functions (i.e., ELU, GELU, and Leaky RELU) are almost the same. The shaded areas are confidence intervals. The bar plots in **c**–**f** are the results of statistical tests that compare the accuracy between the model trained with a candidate method and the original model at the corresponding noise level (*σ* = 0–1.5). If the difference is statistically significant, the color will be blue (*p*_value_ ≤ 0.05, smaller *p* value indicates larger significance), if not, the color will be white.

A DNN can be regarded as a composition of many nonlinear functions. Given input data $x\in {{{{{{{{\mathcal{R}}}}}}}}}^{d}$ and its corresponding label *y*, the DNN aims to minimize the loss $\ell \left({f}_{\theta }(x),y\right)$, where *ℓ* is the loss function, and *f*_*θ*_ is the neural network with drift-inducing parameters *θ*. As shown in Fig. 1a, analog noise such as thermal noise, programming errors, and manufacturing errors will add drift-inducing parameters *θ* to the neural network that affects the robustness of the DNN. This can compromise the precision of machine learning models. In Fig. 1b, the decision boundary for a two-class classification problem shifts towards the wrong side with the increase of parameter drifting (*σ*). Instead of proposing new materials or optimizing circuitry design to improve the robustness of the system to analog noise, we consider forging a DNN that is self-immuned to analog noise without any additional hardware modifications. To systematically analyze the effect of DNN architectural choices on the robustness of analog noise, we first implemented a memristor simulator, MemSim, to analyze the impact of different factors on the performance of analog DNNs. The implementation details are presented in the Method section. Four possible influential factors including inductive noise, normalization, model complexity, and activation functions were examined by Ablation studies to determine the key factors affecting the robustness of analog DNNs. The following list discusses the influence of each factor in detail, and the results are shown in Fig. 1c–f, using the MNIST classification task as an example. We also include results on other memristor simulation platforms such as MemTorch and IBM-aihwkit to test the applicability of our approach on different devices (Supplementary Note 1 and Figs. S1, S2). Our method exhibits consistent and statistically significant improvements over the baseline method on different analog computing hardware. This shows that our approach is practical, can be universally deployed, and does not require any modification to the hardware.

- **Inductive noise:** the influence of adding dropout/noise was examined to be positive for the robustness of the analog DNN, as illustrated in Fig. 1c. The network with dropout layers could gain self-healing ability when parameters are randomly removed during the training process. Therefore, the improvement of robustness to missing parameters strengthens the immunity of the network to parameter drifting.
- **Normalization:** it is observed in Fig. 1d that adding normalization, regardless of the type, deteriorates the performance of the analog DNN. This is because the parameter drifting on the scaling factors in normalization amplifies the drifting effect on the network accuracy.
- **Model complexity and activation functions:** contrary to the common assumption in machine learning that complex DNNs are more robust, increasing the complexity of an analog DNN reduces its robustness due to the errors caused by the accumulation of parameter drifting as the layers go deeper (Fig. 1d). Finally, the effects of other factors, such as the activation function shown in Fig. 1e are negligible for the robustness of the analog DNN.

Though adding dropout layers can significantly improve the robustness of the analog DNN to parameter drifting, they can also cause suboptimal performances due to misspecifications. Therefore, it is crucial to automatically search for an optimal neural network architecture with proper noise injection settings to ensure robustness while avoiding uncertainties. To simplify the neural architecture search, instead of looking for all possible topology structures of the DNN^15–17^, we appended noise injection layers after each DNN layer except the last softmax layer for output, and we searched for the dropout rate of each layer only. We denote the specification of the additional noise injection layers as $\alpha \in {{{{{{{{\mathcal{R}}}}}}}}}^{K-1}$, where *K* is the number of layers in the DNN. The advantage of this neural architecture search space design is not only its simplicity, but also its strong compatibility with all existing neural network architectures. The effectiveness of noise injection is proved with a functional optimization framework as demonstrated in Supplementary Note 2 and Fig. S3. Intuitively, noise injection randomly samples the landscape of the neural network parameter space in analog DNNs. Compared to single-point optimization, noise-injected optimization is equivalent to optimizing the sampled region of neural network parameters. This can expand the parameter space of the robust neural network centered on the original unperturbed parameters during optimization, adding an allowable perturbation range within which the performance of the algorithm would not change. In other words, if the randomized analog DNN gives a correct prediction, for any perturbation on the neural network parameters within the allowable range, the DNN can still yield the same performance. According to the theoretical analysis, the type (spectrum) of the injected noise determines the positions of sampling around the original parameter point and therefore the radius of the robust region of analog DNNs. We examined three different types of noise, including Binomial distribution (also known as dropout), Gaussian distribution, and Laplace distribution, to theoretically explain their effectiveness in robustness improvement and to identify their allowable ranges, respectively (Supplementary Note 2). In the proof, we further revealed the complex trade-off between robustness against analog noise and accuracy. This motivates us to employ Bayesian optimization to determine the optimal setting of the noise level (*α*). Because the noise levels are not differentiable, we choose Bayesian optimization as it does not require the gradients for variables to be optimized and the implementation details are presented in the Methods section.

### Discussions on the effectiveness of BayesFT

We then examine the effectiveness of our method by performing image recognition experiments on several datasets including the Modified National Institute of Standards and Technology (MNIST, ref. ^18^) dataset, the Canadian Institute for Advanced Research-10 (CIFAR-10, ref. ^19^) dataset, the German Traffic Sign Recognition Dataset (GTSRB, ref. ^20^), the Penn-Fudan Database for Pedestrian Detection (PennFudanPed, ref. ^21^), and the Karlsruhe Institute of Technology and Toyota Technological Institute at Chicago Vision Benchmark (KITTI, ref. ^22^). For comparison, we implemented the following state-of-the-art baseline algorithms as references: Empirical risk minimization (ERM) which is the baseline algorithm that only minimizes the empirical risk; ReRAM-variations (ReRAM-V, ref. ^6^) which diagnoses the ReRAM circuits and iteratively re-tunes the parameters to improve robustness to parameter drift until BayesFT converges; Adversarial weight perturbations (AWP, ref. ^23^) which adversely trains the neural network against parameter perturbations to improve robustness to parameter shifts; and Fault-tolerant neural network architecture (FTNA, ref. ^1^) which replaces the last softmax layer of the original model with an error-correction coding scheme as discussed before. We denote our method as BayesFT-DO, BayesFT-Ga, and BayesFT-La, corresponding to different types of injecting noise, namely Bernoulli noise, Gaussian noise, and Laplace noise, respectively.

**Fig. 2:**
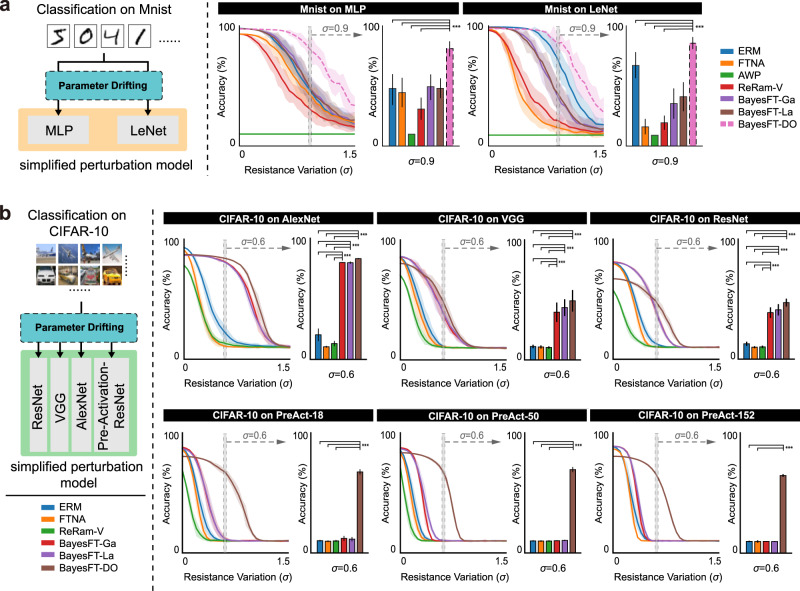
Experimental results on image classification tasks. **a** Experiment results on the MNIST dataset. **b** Experiment results on the CIFAR-10 dataset. The left part in each panels **a**, **b** is a schematic demonstration of the task itself. The results are presented by curve charts and bar charts. The curve chart compares the prediction accuracy of our methods (BayesFT-Ga, BayesFT-La, and BayesFT-Do) and the baseline methods (ERM, FTNA, AWP, and ReRam-V) at different resistance variation (*σ* = 0–1.5). The shaded areas are confidence intervals. The bar charts with confidence intervals show the results of statistical tests (i.e., run the task 20 times and compare the accuracy) at a specific *σ* setting. The horizontal line above the bars indicates the statistical difference in the performance of our methods compared to the baseline methods (if the difference is significant, it will be marked by the *** symbol. Otherwise, the *p* value will be displayed). Specific *σ* values are selected as they can present challenging environments.

**Fig. 3:**
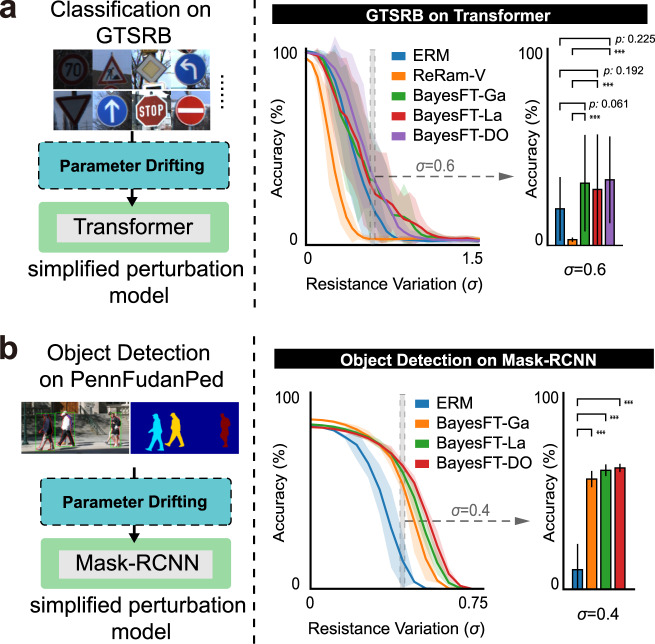
Experiments on image classification and 2D object detection. Experimental results on **a** traffic sign classification on the GTSRB dataset, **b** object detection on the PennFudanPed dataset. The left part in each panel **a**, **b** is a schematic demonstration of the task itself. The results are presented by curve charts and bar charts. The curve chart compares the prediction accuracy of our methods (BayesFT-Ga, BayesFT-La, and BayesFT-Do) and the baseline methods (ERM and ReRam-V) at different resistance variations (*σ* = 0–1.5). The shaded areas are confidence intervals. The bar charts with confidence intervals show the results of statistical tests (i.e., run the task 20 times and compare the accuracy) at a specific *σ* setting. The horizontal line above the bars indicates the statistical difference in the performance of our methods compared to the baseline methods. (if the difference is significant, it will be marked by the *** symbol. Otherwise, the *p* value will be displayed.) Specific *σ* values are selected as they can present challenging environments.

Each algorithm was run 20 times on the MemSim simulation platform, and the mean (line) and standard deviation (shaded area) of accuracy under different resistance variations are demonstrated in Figs. 2, 3. The performance of the system was first evaluated on MNIST^18^. The experiments were carried out on a three-layer multilayer perceptron (MLP) and LeNet5^24^ with drifting terms (resistance variation, *σ*: 0–1.5) applied as the noise level to the model (Fig. 2a). All algorithms except BayesFT-DO exhibit severe accuracy degradation with increasing resistance variation. For the classification of MLP, the accuracy of BayesFT-DO remains constant within the small variance region (*σ* ≤ 0.6) and only drops slightly when *σ* exceeds 0.9. In contrast, the other algorithms exhibit significant accuracy dropping when *σ* reaches 0.2. The accuracy values for all algorithms at *σ* = 0.9 are provided in Fig. 2a for a more intuitional comparison. BayesFT-DO also out-competes the other algorithms in terms of the mean accuracy, with at least a 30% improvement (*p* < 0.001, two-sided *t*-test). A similar trend can be observed in LeNet, where BayesFT-DO outperforms other baseline methods. This is in good agreement with our theoretical analysis (Supplementary Note 2) that adding Bernoulli distributed noise (dropout) can improve the fault tolerance of DNNs to parameter drifting.

A similar experimental protocol was applied to the CIFAR-10 dataset with various neural network architectures, including the most commonly used ones in computer vision, such as AlexNet^25^, VGG^26^, ResNet^27^, and PreAct-ResNet (PreAct, ref. ^27^) with different numbers of layers. Compared with handwritten digits in the MNIST dataset, CIFAR-10 contains real-world objects that pose more difficulties for recognition. The results are shown in Fig. 2b. The classification accuracy of the baseline methods decreases rapidly as the resistance variance increases, while BayesFT optimization still has stable performance. For example, on AlexNet, the accuracy of BayesFT-DO remains almost the same for *σ* < 0.6, and it drops by only 20% for *σ* < 0.8. The accuracy improvement obtained by BayeFT-DO increases from 17 to 68% compared to ERM when *σ* is varied from 0.3 to 0.9. BayesFT-DO also demonstrates competitive results on VGG and ResNet architectures. It is worth noting that on VGG, ResNet, and AlexNet, the performances of BayesFT-Ga and BayesFT-La are rather close to BayesFT-DO and out-compete other baseline methods. Stronger superiority of BayesFT can be observed on the PreAct-18, PreAct-50, and PreAct-152 networks showing its much greater insensitivity to high-level noise (Fig. 2b lower panels). Recognition results from other realistic tasks, including traffic sign recognition (Fig. 3a) and pedestrian detection (Fig. 3b), provide extra evidence for the effectiveness of BayesFT in improving the robustness of analog DNNs. For example, compared with the baseline method ERM, BayesFT achieves three times the accuracy at *σ* = 0.4, largely improving the safety of pedestrian detection systems on analog computing devices.

**Fig. 4:**
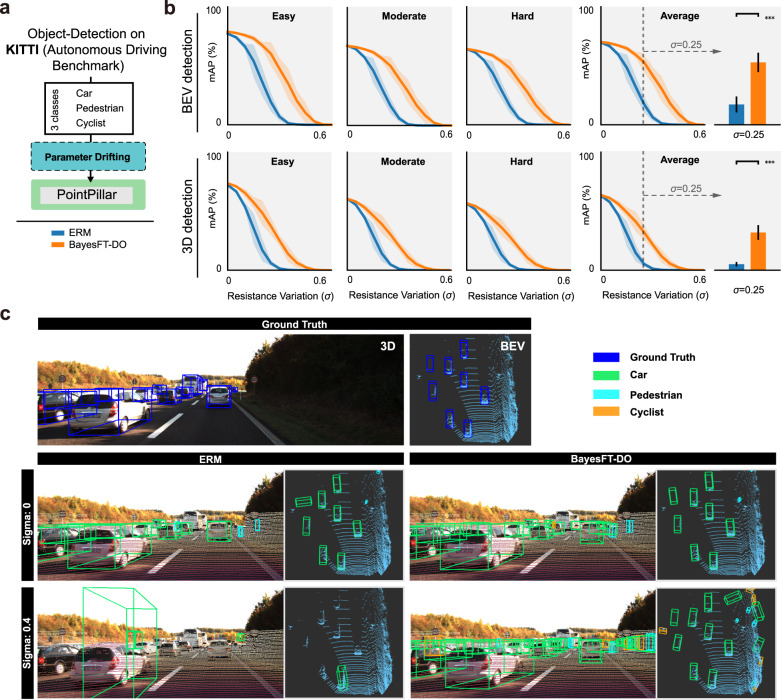
Experimental results of 3D object detection in autonomous driving tasks. **a** The experimental setting of the object detection task on KITTI. Three types of objects were detected: cars, pedestrians, and cyclists. **b** Numerical results of the KITTI experiment. The blue curves are the results of the baseline method. The yellow curves are the results of our approach. The proposed method can achieve more than 100 times better performance under large resistance variances on all KITTI dataset subsets (Easy, Moderate, and Hard). The shaded areas are confidence intervals. The bar charts on the right with confidence intervals show the results of statistical tests (i.e., run the task for 20 times and compare the accuracy) at *σ* = 0.25. The horizontal line above the bars indicates the statistical difference in the performance of our methods compared to the baseline methods (if the difference is significant, it will be marked by the *** symbol. Otherwise, the *p* value will be displayed). **c** Visualization of 3D object detection results. The top figure is the Bird’s Eye View of the ground truth detection result. The left bottom figure is the baseline method’s result. The bottom right figure is our approach’s result. Cars and cyclists are not detected by baseline methods, while the proposed method can successfully detect these objects. The safety of analog DNNs can be largely improved with our approach.

We finally conducted experiments on an autonomous driving task of point cloud detection with the KITTI^28^ dataset to detect cars, pedestrians, and cyclists on the road, in which data is collected by a Velodyne Lidar. A point cloud is a bunch of points that contains the location information of each point in three-dimensional space^29^, and it plays an essential role in many contemporary autonomous driving systems. We compared the performance of different training algorithms using the PointPillars networks^30^, a fast DNN model for object detection from point clouds. Similar to the above experiments, perturbations with *σ* from 0 to 0.6 were applied as the drift-inducing parameters for the trained model (Fig. 4a). Two typical 3D object detection metrics, Bird’s Eye View (BEV) and 3D Detection, were evaluated to examine the performance of the baseline method (ERM) and our method. As shown in Fig. 4b, BayesFT-DO outperforms ERM in terms of the mean average precision (mAP) over the entire perturbation *σ* range for both BEV and 3D detection. This means that BayesFT, compared to ERM, can provide more accurate recognition in both noise-free and noise-injected environments. The visualized results shown in Fig. 4c are in good agreement with this conclusion. Both the baseline and our methods can achieve good performance when *σ* is zero. However, when *σ* reaches 0.4, the recognition accuracy of ERM decreases sharply without even one car being detected correctly, while BayesFT-DO can still detect most of the cars accurately. Consequently, from the experimental results, the proposed method, namely BayesFT-DO, could improve the robustness of analog DNNs, especially for critical applications, such as autonomous driving, and improve the recognition accuracy even in a noisy environment.

## Conclusion

To conclude, in this work, in contrast to the previous efforts which focus on improving the accuracy of analog DNNs from a device manufacturing perspective, we provide a train-time optimization framework to improve the robustness of analog DNNs to achieve accurate recognition without any hardware modification. By systematically analyzing the influence of different factors on the performance of analog DNNs through a memristor simulator, we find that injecting noise can efficiently improve the robustness, for which we also provide theoretical proof. BayesFT was applied to optimize the setting and distribution of the injected noise, and its working principle was proved theoretically, making it the first theoretically guaranteed method for training robust analog DNNs. BayesFT is generalizable to various DNN architectures and its effectiveness was examined by different recognition tasks, including image classification (MNIST and CIFAR-10), traffic sign recognition, and 3D point detection (KITTI). BayesFT makes the analog DNN insensitive to noise while inducing only relatively low accuracy loss, even at high-level noise. We believe our findings could extend the practicability of analog DNNs to previously impossible life-critical tasks, such as autonomous driving, by providing both good empirical performances and theoretical guarantees.

## Methods

### Implementation details of MemSim

For simplicity and to avoid loss of generality, we adopt a memristor perturbation model following a challenging setting as used in refs. ^6, 31^. This model is fitted on real devices and considers multiple factors resulting in the memristance drifting, including thermal noise, programming errors, and manufacturing errors. Specifically, the drifting term is applied to each neural network parameter *θ*_*i*_:

1 $${\theta }_{i}^{{\prime} }\leftarrow {\theta }_{i}{e}^{\lambda },\quad \lambda \sim {{{{{{{\mathcal{N}}}}}}}}(0,{\sigma }^{2})$$

where ${\theta }_{i}^{{\prime} }$ is the drifted neural network parameters, which follow a log-normal distribution. By changing the *σ* value, we can simulate different perturbation levels under various devices and deployment scenarios. The example Pytorch code of the simulator is shown as follows (more simulation platform results with different device models can be seen in Supplementary Note 1):

import torch


import numpy as np


”’

*model: Python code for neural network architecture;*

*model_path: Saved parameters for neural networks;*

*valid_dl: Data loader for validation dataset*.

”’


def evaluate_MemSim_Robustness(model, model_path, valid_dl):


*# Pick different sigma values*

sigma= np.linspace(0., 1.5, 31)

*# Initialize an empty list for accuracy under different sigma values*

accu = []

*# Run several times for statistical tests*

num = 20

evaluated = np.zeros(num)

for std in sigma:

for i in range(num):

model.load_state_dict(torch.load(model_path))

add_noise_to_parameters(0, std, model)

*#Add noise to the parameters of neural networks*

evaluated[i] = evaluate_accuracy(model, valid_dl)[’val_acc’]

accu.append(np.sum(evaluated)/num)

return sigma, accu

### Implementation details of optimization methods

We first define our objective function by marginalizing the loss over the distribution of drifting neural network parameters ***θ***:

2 $$u({{{{{{{\boldsymbol{\alpha }}}}}}}},{{{{{{{\boldsymbol{\theta }}}}}}}})=-{{\mathbb{E}}}_{\tilde{{{{{{{{\boldsymbol{\theta }}}}}}}}} \sim p(\tilde{{{{{{{{\boldsymbol{\theta }}}}}}}}})}[\ell ({f}_{({{{{{{{\boldsymbol{\alpha }}}}}}}},\tilde{{{{{{{{\boldsymbol{\theta }}}}}}}}})}({{{{{{{\boldsymbol{x}}}}}}}}),{{{{{{{\boldsymbol{y}}}}}}}})]$$

where $\tilde{{{{{{{{\boldsymbol{\theta }}}}}}}}}={{{{{{{\boldsymbol{\theta }}}}}}}}\exp (\lambda )$; $\lambda \sim {{{{{{{\mathcal{N}}}}}}}}(0,{\sigma }^{2})$, $\ell ({f}_{({{{{{{{\boldsymbol{\alpha }}}}}}}},\tilde{{{{{{{{\boldsymbol{\theta }}}}}}}}})}({{{{{{{\boldsymbol{x}}}}}}}}),{{{{{{{\boldsymbol{y}}}}}}}})$ is the loss of a neural network with the noise setting ***α*** (*e.g*. dropout rates for Binomial noises) and parameter ***θ*** given input data ***x*** and target ***y***. This intractable equation can be approximately computed by Monte Carlo sampling:

3 $$u({{{{{{{\boldsymbol{\alpha }}}}}}}},{{{{{{{\boldsymbol{\theta }}}}}}}})\simeq -\frac{1}{T}\mathop{\sum }\limits_{t=1}^{T}\ell \left.({f}_{({{{{{{{\boldsymbol{\alpha }}}}}}}},{\tilde{{{{{{{{\boldsymbol{\theta }}}}}}}}}}_{t})}({{{{{{{\boldsymbol{x}}}}}}}}),{{{{{{{\boldsymbol{y}}}}}}}})\right]$$

where *T* is the number of Monte Carlo samples and ${\tilde{{{{{{{{\boldsymbol{\theta }}}}}}}}}}_{t}$ is the *t*-th sample randomly drawn from the distribution of parameter $p(\tilde{{{{{{{{\boldsymbol{\theta }}}}}}}}})$. To maximize the objective function, we use an optimization scheme where ***α*** and ***θ*** are optimized alternatively. When optimizing ***α***, as discussed in the main text, because there is no exact gradient information available for ***α***, we use Bayesian optimization, which does not require the gradients for variables to be optimized. Bayesian optimization uses a surrogate model constructed from previous trials to determine the point for the next trial, i.e. the point which is the most likely to give the optimal solution for the gradient-free optimization problem^32^. For ***θ***, we employ the stochastic gradient descent method.

In terms of Bayesian optimization, we use a Gaussian process regression model as the surrogate model. Suppose we already have *n* trials of different settings of ***α*** denoted as ***α***_1:*n*_, its corresponding objective function value *g*(***α***_1:*n*_), and kernel matrix *κ*(***α***_1:*n*_, ***α***_1:*n*_), more specifically:

4 $${{{{{{{{\boldsymbol{\alpha }}}}}}}}}_{1:n}=[{{{{{{{{\boldsymbol{\alpha }}}}}}}}}_{1},\cdots \,,{{{{{{{{\boldsymbol{\alpha }}}}}}}}}_{n}]$$

5 $$g({{{{{{{{\boldsymbol{\alpha }}}}}}}}}_{1:n})=[u({{{{{{{{\boldsymbol{\alpha }}}}}}}}}_{1},{{{{{{{\boldsymbol{\theta }}}}}}}}),\cdots \,,u({{{{{{{{\boldsymbol{\alpha }}}}}}}}}_{n},{{{{{{{\boldsymbol{\theta }}}}}}}})]$$

6 $$\kappa ({{{{{{{{\boldsymbol{\alpha }}}}}}}}}_{1:n},{{{{{{{{\boldsymbol{\alpha }}}}}}}}}_{1:n})=\left[\begin{array}{ccc}\kappa ({{{{{{{{\boldsymbol{\alpha }}}}}}}}}_{1},{{{{{{{{\boldsymbol{\alpha }}}}}}}}}_{1}),&\cdots \,,&\kappa ({{{{{{{{\boldsymbol{\alpha }}}}}}}}}_{1},{{{{{{{{\boldsymbol{\alpha }}}}}}}}}_{n})\\ \cdots \,,&\cdots \,,&\cdots \\ \kappa ({{{{{{{{\boldsymbol{\alpha }}}}}}}}}_{n},{{{{{{{{\boldsymbol{\alpha }}}}}}}}}_{1}),&\cdots \,,&\kappa ({{{{{{{{\boldsymbol{\alpha }}}}}}}}}_{n},{{{{{{{{\boldsymbol{\alpha }}}}}}}}}_{n})\end{array}\right]$$

Then, according to the Gaussian process’s property, the posterior probability of *g*(***α***) after *n* trials follows a Gaussian distribution: where *κ* is the kernel function. In our experiment, we use the exponential kernel function:

7 $$\kappa ({{{{{{{{\boldsymbol{\alpha }}}}}}}}}_{1},{{{{{{{{\boldsymbol{\alpha }}}}}}}}}_{2})={k}_{0}\exp (-\parallel {{{{{{{{\boldsymbol{\alpha }}}}}}}}}_{1}-{{{{{{{{\boldsymbol{\alpha }}}}}}}}}_{2}{\parallel }^{2})$$

where $\parallel {{{{{{{{\boldsymbol{\alpha }}}}}}}}}_{1}-{{{{{{{{\boldsymbol{\alpha }}}}}}}}}_{2}{\parallel }^{2}=\mathop{\sum }\nolimits_{i = 1}^{d}{k}_{i}{({\alpha }_{1,i}-{\alpha }_{2,i})}^{2}$, and *k*_*i*_ are parameters of the kernel.

Then, the next trial is given by finding the point that is most likely to give the optimal objective value: ${{{{{{{{\boldsymbol{\alpha }}}}}}}}}^{* }=\mathop{\max }\nolimits_{{{{{{{{{\boldsymbol{\alpha }}}}}}}}}^{* }}p(g({{{{{{{{\boldsymbol{\alpha }}}}}}}}}^{* })| g({{{{{{{{\boldsymbol{\alpha }}}}}}}}}_{1:n}))$. Based on the above theory, we generate the algorithm based on Bayesian optimization for fault-tolerant neural network architecture, as shown in Algorithm 1.

#### Algorithm 1

**Bayesian Optimization for Fault-Tolerant DNN (BayesFT)**

**Input:** Dataset (***x***, ***y***), neural network parameters ***θ***, dropout rates for each layer ***α***, number of epochs for training neural networks *E*.

**Output:** Trained neural network ***θ*** and dropout rates for each layer ***α***.

**Initialization:** initialize ***θ*** with Xavier random initialization^33^, ***α*** with a uniform distribution on [0, 1], number of iterations *t* = 0:

**repeat**

**for** *e* = 1 to *e* = *E* **do**

Optimize neural network parameters ***θ***:

$${{{{{{{{\boldsymbol{\theta }}}}}}}}}_{t}\leftarrow {{{{{{{{\boldsymbol{\theta }}}}}}}}}_{t-1}-{\nabla }_{{{{{{{{\boldsymbol{\theta }}}}}}}}}u({\alpha }_{t-1},{{{{{{{{\boldsymbol{\theta }}}}}}}}}_{t-1})$$

**end for**

Update the posterior distribution function for Bayesian optimization:

$$g({{{{{{{{\boldsymbol{\alpha }}}}}}}}}_{1:t-1})=[u({{{{{{{{\boldsymbol{\alpha }}}}}}}}}_{1},{{{{{{{{\boldsymbol{\theta }}}}}}}}}_{t}),\cdots \,,u({{{{{{{{\boldsymbol{\alpha }}}}}}}}}_{t-1},{{{{{{{{\boldsymbol{\theta }}}}}}}}}_{t})]$$

$${u}_{t-1}({{{{{{{\boldsymbol{\alpha }}}}}}}})=\kappa ({{{{{{{\boldsymbol{\alpha }}}}}}}},{{{{{{{{\boldsymbol{\alpha }}}}}}}}}_{1:t-1})\kappa {({{{{{{{{\boldsymbol{\alpha }}}}}}}}}_{1:t-1},{{{{{{{{\boldsymbol{\alpha }}}}}}}}}_{1:t-1})}^{-1}g({\alpha }_{1:t-1})$$

$${\sigma }_{t-1}^{2}({{{{{{{\boldsymbol{\alpha }}}}}}}})=\kappa ({{{{{{{\boldsymbol{\alpha }}}}}}}},{{{{{{{\boldsymbol{\alpha }}}}}}}})-\kappa ({{{{{{{\boldsymbol{\alpha }}}}}}}},{{{{{{{{\boldsymbol{\alpha }}}}}}}}}_{1:t-1})\kappa {({{{{{{{{\boldsymbol{\alpha }}}}}}}}}_{1:t-1},{{{{{{{{\boldsymbol{\alpha }}}}}}}}}_{1:t-1})}^{-1}\kappa ({{{{{{{{\boldsymbol{\alpha }}}}}}}}}_{1:t-1},{{{{{{{\boldsymbol{\alpha }}}}}}}})$$

Calculate the optimal ***α*** from the updated posterior distribution function for the surrogate model:

$${{{{{{{{\boldsymbol{\alpha }}}}}}}}}_{t}\leftarrow \mathop{\max }\limits_{{{{{{{{\boldsymbol{\alpha }}}}}}}}}p(g({{{{{{{\boldsymbol{\alpha }}}}}}}})| g({{{{{{{{\boldsymbol{\alpha }}}}}}}}}_{1:t-1}))$$

*t* ← *t* + 1

**until** convergence;

### Supplementary information

Supplementary Information

## Data Availability

The data supporting the results of this work are available from the corresponding author upon reasonable request.

## Code availability

Code for this article is available from the corresponding author upon reasonable request.
