# Supplementary material for: Improving the robustness of analog deep neural networks through a Bayes-optimized noise injection approach
Source: Commun Eng. 2023 May 11;2:25. doi: 10.1038/s44172-023-00074-3 (PMC10956073; doi:10.1038/s44172-023-00074-3)
Supplement: Supplementary file 1 — Supplementary Information [file 44172_2023_74_MOESM1_ESM.pdf]

# Supplementary Information for Improving the Robustness of Analog Deep Neural Networks Through a Bayes-optimized Noise Injection Approach

# Supplementary Note 1: Results on Other Circuit Simulation Platforms

## 1.1 Results of ReRAM devices

In addition to the ReRAM drifting model used in the paper, we have extended the evaluation on other circuit simulation platforms to model the non-idealities of the memristor to show that our method can be generalized to different kinds of noise regardless of the hardware implementation details. Specifically, we deployed the baseline algorithms and our BayesFT optimization methods on *MemTorch* [1]. This is an open-source simulation platform for memristive deep learning systems, which integrates directly with the PyTorch library. In this simulation, we used the default setting of 8-bit ADC resolution and the quantization method is linear.

In the simulation, we used the Voltage ThrEshold Adaptive Memristor (VTEAM) model [2], which is a general and accurate model to describe the behavior of voltage-controlled memristors.

In order to better demonstrate the generalization of our BayesFT method, we selected three different groups of parameters in [2] for the experiment, which correspond to three kinds of experimental devices, namely Pt-Hf-Ti memristor [3], ferroelectric memristor [4], and metallic nanowire memristor [5].

The actual $\hat{R}_{on}$ and $\hat{R}_{off}$ parameters in the simulation software are sampled stochastically from a normal distribution, with a mean value of $R_{on}$ and $R_{off}$ in the reference parameters, and a variance $\sigma$ which can be changed. As $\sigma$ increases, the device-device variability increases, and the inference accuracy of an analog neural network decreases. By comparing the degree of accuracy degradation at the same variance, we can compare the performance of different algorithms.

Based on the above experimental settings, we applied our BayesFT optimization method and baseline algorithms to classify MNIST with LeNet, because convolutional neural networks are used more often in image classification tasks.

As shown in Fig. S1 (b), the results for the three different settings show the same trend. The accuracy of BayesFT-DO shows great robustness, while the accuracy of FTNA drops rapidly as the resistance variance increases. The performance of ERM and ReRAM-V is intermediate between the BayesFT method and the FTNA method. Note that the $R_{on}$ and $R_{off}$ values of the three settings are of different orders of magnitude, so the scales of $\sigma$, i.e., the scale of the horizontal axis, is different.

## 1.2 Results of PCM devices

In addition to ReRAM, our method also works on phase-change memory (PCM) devices. We conducted experiments on the *IBM Analog Hardware Acceleration Kit*[6](*IBM-aihwkit*), which provides state-of-the-art statistical models of PCM arrays that can be used to simulate various sources of noise present in real hardware when performing inference.

The model simulated three different sources of noise from the PCM array: programming noise, read noise, and temporal drift. After training the network in software, the trained parameters are mapped to target conductance values $G_T$. The conductance values are then programmed on a PCM device in hardware using a closed-loop iterative write-read-verify scheme. There will be a difference between $G_T$ and the actual conductance value $G_{prog}$, which is characterized by the programming noise. The programming noise is described by (1) and (2).

$$g_{prog} = g_T + \mathcal{N}(0, \sigma_{prog}) \tag{1}$$

$$\sigma_{prog} = \max\left(-1.1731 g_T^2 + 1.9650 g_T + 0.2635, 0\right) \tag{2}$$

After the parameters have been programmed, the conductance value of the PCM device drifts with time. Knowing the conductance at time $t_c$ of the last programming pulse, $g_{prog}$, the conductance at time $t$ is:

$$g_{drift}(t) = g_{prog}\left(\frac{t}{t_c}\right)^{-\nu} \tag{3}$$

where $\nu$ is sampled from $\mathcal{N}(\mu_\nu, \sigma_\nu)$.

Read noise occurs when the devices are read after they have been programmed. The standard deviation of the read noise $\sigma_{nG}$ at time $t$ is:

$$\sigma_{nG}(t) = g_{drift}(t) Q_s \sqrt{\log \frac{t + t_{read}}{2t_{read}}} \tag{4}$$

where $t_{read} = 250\text{ns}$ is the pulse width applied when reading the devices. $Q_s$ measured from the PCM device as a function of $g_T$ is given by:

$$Q_s = \min\left(0.0088/g_T^{0.65}, 0.2\right) \tag{5}$$

The final simulated PCM conductance from the model at time $t$ is given by:

$$g(t) = g_{drift}(t) + \mathcal{N}(0, \sigma_{nG}(t)) \tag{6}$$

The noise and drift described above are long-term non-linearities. Besides that, the parameter noise, which is short-term noise, is applied for each MAC computation, while not touching the actual parameter matrix but referring it to the output:

$$y_i = \sum_j w_{ij} + \xi_{ij} \tag{7}$$

where $\xi \sim \mathcal{N}(0, \sigma)$, thus the parameters are all Gaussian distributed. $\sigma$ is determined by *w_noise*.

We deployed the baseline algorithm ERM and our method BayesFT-DO, BayesFT-Ga, and BayesFT-La on *IBM-aihwkit*. We evaluated different neural network architectures including LeNet and three-layer MLP on MNIST, and AlexNet, VGG11, ResNet18, and Preact-ResNet with different numbers of layers on CIFAR-10. Different *w_noise* was applied for all groups of experiments. The results are shown in Fig. S2.

For all eight sets of experiments, we can observe that the accuracy of BayesFT-DO decreases more slowly than ERM, BayesFT-Ga, and BayesFT-La as *w_noise* increases, indicating that BayesFT-DO yields the best robustness. For example, on MLP, when *w_noise* increases to 0.4, the accuracy of ERM decreases to 49.57%, while

the accuracy of BayesFT-DO remains as high as 73.33%. On AlexNet, when *w_noise* increases to 0.01, compared to the ERM in which the accuracy drops to 32.37%, the accuracy of BayesFT-DO is still as high as 75.80%.

In the CIFAR-10 experiments, BayesFT-Ga and BayesFT-La achieve slightly higher accuracy than ERM. For VGG11, BayesFT-Ga and BayesFT-La show much better robustness than ERM. When *w_noise* increases to 0.02, the accuracy of ERM decreases to 15.81%, while the accuracy of BayesFT-DO is 77.49%, BayesFT-Ga is 71.63%, and BayesFT-La is 72.20%. This is because the VGG11 network structure with batch normalization is used in the ERM method, while our methods remove most of the normalization layers, thus improving the robustness.

# Supplementary Note 2: Theoretical proof of robustness

In this section, before we dive into the mathematical proof, we first introduce an intuitive example as illustrated in Figure S3 of the basic idea of our method. The aim is to prove that there exists a region around the original parameter point where the loss value is upper bounded by a constant, or equivalently the *likelihood* value is lower bounded by a constant. Due to the highly non-convex property of the likelihood function landscapes with regard to parameters $\theta$ in the DNN, we take a different approach that does not rely on strong convexity assumptions of the DNN. Considering a randomized version of the analog DNN with injected noise to actively perturb the original parameter, by increasing the number of injected random noise samples, the worst possible likelihood value can be determined with higher confidence via concentration inequalities. With no assumptions related to the shape of the likelihood functional, the likelihood functional has to travel through all sampled points to ensure the consistency of our random measurement. With this constraint on the likelihood functional, we can then conclude that within a closed set around the original parameter, the analog DNN can maintain the correct output (i.e. above the lower bounded constant). Therefore, as long as the magnitudes of the analog noise are within some ranges, the robustness of the analog DNN can be guaranteed. This indicates that analog DNN can be equivalent to digital DNN under some conditions, which could enables analog DNN to be deployed for life-critical systems. Besides, during the DNN optimization phase with injected noise layers, we optimize on a region centered on the original parameter instead of a single parameter, which can improve the generalization ability of analog DNN by avoiding sharp minima.

In the following part, we provide a formal theoretical justification of the proposed algorithm. We first introduce the proposed randomized version of the analog DNN model: $f_{\pi_0}(\theta_0, x) := \mathbb{E}_{\eta \sim \pi_0}[f(\theta_0 \otimes \eta, x]$, where $x$ is the input data; $\theta_0$ is the original unperturbed parameters of the analog DNN; $\pi_0$ is the random noise injected in the analog DNN; $\otimes$ is the operator for applying the random distribution, which can be $+$ for Gaussian distribution or $\times$ for Dropout. In the following parts, we write $f_{\pi_0}(\theta_0, x)$ as $f_{\pi_0}(\theta_0)$ for brevity. For simplicity and avoiding loss of generality, we consider the case of a 0-1 classification

problem. We want to prove that if $f_{\pi_0} > \frac{1}{2}$, then for any perturbation on the analog DNN's parameter $\delta$ within some ranges, the prediction of the analog DNN remains the same (i.e. $f_{\pi_0}(\theta_0 \otimes \delta) > \frac{1}{2}$).

To derive a tractable lower bound for $f_{\pi_0}(\theta_0 \otimes \delta)$ and deduce $\delta$, we relax $f$ in the functional space $\mathcal{F} = \{\hat{f} : \hat{f}(\theta) \in [0, 1]\}$ which is the set of all functions bounded in $[0, 1]$, along with a equality constraint at the original function $f$:

$$\begin{aligned} &\min_{\delta \in \mathcal{B}} f_{\pi_0}(\theta_0 \otimes \delta) \geq \min_{\hat{f} \in \mathcal{F}} \left\{ \min_{\delta \in \mathcal{B}} \hat{f}_{\pi_0}(\theta_0 \otimes \delta) \right\} \\ &\text{s.t.} \;\; \hat{f}_{\pi_0}(\theta_0) = f_{\pi_0}(\theta_0) \end{aligned} \tag{8}$$

**Theorem 1** ***(Lagrangian)*** *Denote by $\pi_\delta$ the distribution of $\eta \otimes \delta$, solving Inequality 8 is equivalent to solving the following problem:*

$$\begin{aligned} \mathcal{L} &= \min_{\hat{f} \in \mathcal{F}} \min_{\delta \in \mathcal{B}} \max_{\lambda \in \mathbb{R}} \left\{ \hat{f}_{\pi_0}(\theta_0 \otimes \delta) - \lambda(\hat{f}_{\pi_0}(\theta_0) - f_{\pi_0}(\theta_0)) \right\} \\ &\geq \max_{\lambda \geq 0} \left\{ \lambda f_{\pi_0}(\theta_0) - \max_{\delta \in \mathcal{B}} \mathbb{D}_{\mathcal{F}}(\lambda \pi_0, \pi_\delta) \right\} \end{aligned} \tag{9}$$

*where $\mathbb{D}_{\mathcal{F}}(\lambda \pi_0, \pi_\delta)$ is:*

$$\begin{aligned} &\mathbb{D}_{\mathcal{F}}(\lambda \pi_0, \pi_\delta) \\ &= \max_{\hat{f} \in \mathcal{F}} \left\{ \lambda \mathbb{E}_{\eta \sim \pi_0}[\hat{f}(\theta_0 \otimes \eta)] - \mathbb{E}_{\eta \sim \pi_\delta}[\hat{f}(\theta_0 \otimes \eta)] \right\} \\ &= \begin{cases} \int [\lambda \pi_0(\eta) - \pi_\delta(\eta)]_+ \mathrm{d}\eta, \textit{ if } \pi \textit{ is continuous,} \\ \sum [\lambda \pi_0(\eta) - \pi_\delta(\eta)]_+, \textit{ if } \pi \textit{ is discrete.} \end{cases} \end{aligned} \tag{10}$$

Theorem 1 is the Lagrangian inequality which can be proved by the min-max theorem. According to Theorem 1, for different distributions of the injected noise, we can instantiate $\pi_0$ as the type of the distribution and derive the maximum allowable perturbation range $\mathcal{B}$ that for any $\delta \in \mathcal{B}$, the prediction performance of analog DNN remains the same. In the following part in Supplementary Notes 2, we examine three different distribution types, that are, Gaussian distribution, dropout (Binomial distribution), and Laplace distribution, and derive $\mathcal{B}$ respectively with mathematical proofs.

### *Gaussian distribution*

In this case, $\pi_0(\eta)$ is a Gaussian distribution: $\mathcal{N}(0, \sigma^2)$; $\otimes$ is instantiated as $+$. Then, for $\|\delta\|_2 \leq r$, the prediction confidence lowerbound is:

$$\mathcal{L} = \min_{\|\delta\|_2 \leq r} \max_{\lambda \geq 0} \left\{ \lambda f_{\pi_0}(\theta_0) - \int [\lambda \pi_0(\eta) - \pi_{\boldsymbol{\delta}}(\eta)]_+ \mathrm{d}\eta \right\} \tag{11}$$

Define $C_\eta = \{\eta : \lambda \pi_0(\eta) \geq \pi_\delta(\eta)\} = \{\eta : \delta^T(\delta - \theta_0) \leq \frac{\|\delta\|^2}{2} + \sigma^2 \ln \lambda\}$ and $\Phi$ to be the cumulative distribution function of standard Gaussian distribution.

Then also define

$$F(\delta,\lambda) := \lambda f_{\pi_0}(\theta_0) - \int [\lambda\pi_0(\eta) - \pi_\delta(\eta)]_+ \mathrm{d}\eta \tag{12}$$

$$= \lambda f_{\pi_0}(\theta_0) - \int_{C_\lambda} [\lambda\pi_0(\eta) - \pi_\delta(\eta)] \mathrm{d}\eta \tag{13}$$

$$= \lambda f_{\pi_0}(\theta_0) - \lambda\Phi\left(\frac{\|\delta\|_2}{2\sigma} + \frac{\sigma\ln\lambda}{\|\delta\|_2}\right) - \Phi\left(\frac{-\|\delta\|_2}{2\sigma} + \frac{\sigma\ln\lambda}{\|\delta\|_2}\right) \tag{14}$$

Note that $F$ is a concave function with respect to $\lambda$, we have:

$$\mathcal{L} = \min_{\|\delta\|_2 \le r} \max_{\lambda \ge 0} F(\delta,\lambda) \tag{15}$$

$$= \min_{\|\delta\|_2 \le r} \Phi\left(\frac{-\|\delta\|_2}{2\sigma} + \frac{\sigma\ln\hat{\lambda_\delta}}{\|\delta\|_2}\right) \tag{16}$$

$$= \min_{\|\delta\|_2 \le r} \Phi\left(\Phi^{-1}(f_{\pi_0}(\theta_0)) - \frac{\|\delta\|_2}{\sigma}\right) \tag{17}$$

$$= \Phi\left(\Phi^{-1}(f_{\pi_0}(\theta_0)) - \frac{r}{\sigma}\right) \tag{18}$$

We have lower bound $\mathcal{L} \ge \frac{1}{2}$ if and only if $r \le \sigma\Phi^{-1}(f_{\pi_0}(\theta_0))$, i.e., the analog DNN is robust within the perturbation range $\mathcal{B} = \{\delta : \|\delta\|_2 \le \sigma\Phi^{-1}(f_{\pi_0}(\theta_0))\}$. This reveals that the robustness to analog noise is determined by two factors: 1)the magnitude of noise injected during training ($\sigma$). 2)the analog DNN's self-healing ability $f_{\pi_0}(\theta_0)$. Larger injected noise might result in a more robust analog DNN but could also affect the accuracy it can achieve.

***Dropout***

In this case, $\pi_0(\eta)$ is instantiated as a Binomial distribution with a probability $p$ of $\eta$ setting to zero; $\otimes$ is set as $\times$. Then, for $\|\delta\|_2 \le r$, the prediction confidence lowerbound is:

$$\mathcal{L} = \max_{\lambda \ge 0} \left\{ \lambda f_{\pi_0}(\theta_0) - \sum_\eta [\lambda\pi_0(\eta) - \pi_\delta(\eta)]_+ \right\}$$

We have $[\lambda\pi_0(\eta) - \pi_\delta(\eta)]_+ > 0$ only if $\pi_0(\eta) > 0$.

$$\mathcal{L} = \max_{\lambda \ge 0} \left\{ \lambda f_{\pi_0}(\theta_0) - \sum_\eta [\lambda\pi_0(\eta) - \pi_{\boldsymbol{\delta}}(\eta)]_+ \right\}$$

$$= \max_{\lambda \ge 0} \left\{ \lambda f_{\pi_0}(\theta_0) - [\lambda - 1]_+ p^\Theta - \lambda(1 - p^\Theta) \right\}$$

$$= f_{\pi_0}(\theta_0) - 1 + p^\Theta \ge \frac{1}{2} \tag{19}$$

Where $\Theta$ is the dimension of analog DNN's parameters. From the above inequality, we can deduce that as long as the dropout rate $p$ is large enough and the randomized version of the analog DNN model have high confidence on the corrected prediction ($f_{\pi_0}(\theta_0)$ is close to 1), we can obtain provable correct prediction results regardless of the analog noise. This explains the high robustness of BayesFT-DO over a range of tasks. In addition, it reveals an inherent trade-off between robustness and accuracy. Selecting higher $p$ values can decrease the discrepancy in the performance between the model deployed on GPU and analog devices, but it could also result in degraded accuracy due to the accumulation of dropped-out neurons.

***Laplace distribution***

In this case, $\pi_0(\eta)$ is a Laplace distribution: Laplace$(0, b)$. Then, for $\|\delta\|_1 \leq r$, the prediction confidence lowerbound is:

$$\mathcal{L} = \max_{\lambda \geq 0} \left\{ \lambda f_{\pi_0}(\theta_0) - \int [\lambda \pi_0(\eta) - \pi_\delta(\eta)]_+ \mathrm{d}\eta \right\} \tag{20}$$

$$= \max_{\lambda \geq 0} \left\{ \lambda f_{\pi_0}(\theta_0) - \int \frac{1}{2b} \exp\left(-\frac{|\eta|}{b}\right)[\lambda - \exp\left(\frac{|\eta| - |\eta + r|}{b}\right)]_+ \mathrm{d}z \right\} \tag{21}$$

Let $\eta_0$ be the solution of $\lambda - \exp\left(\frac{|\eta|-|\eta+r|}{b}\right) = 0$, then we have

$$\eta_0 = \begin{cases} -\infty, & b \log \lambda \geq r \\ -\frac{1}{2}(b \log \lambda + r), & -r < b \log \lambda < r \\ +\infty, & b \log \lambda \leq -r \end{cases} \tag{22}$$

So the lower bound is

$$\max_{\lambda \geq 0} \left\{ \lambda f_{\pi_0}(\theta_0) - \lambda \int_{\eta_0}^{\infty} \frac{1}{2b} \exp\left(-\frac{|\eta|}{b}\right) \mathrm{d}\eta + \int_{\eta_0}^{\infty} \frac{1}{2b} \exp\left(\frac{|\eta| - |z + r|}{b}\right) \mathrm{d}\eta \right\} \tag{23}$$

For the above three cases, we have:

1. $b \log \lambda \geq r$
   the bound is

$$\max_{\lambda \geq \exp\left(\frac{r}{b}\right)} \{\lambda f_{\pi_0}(\theta_0) - \lambda + 1\} = 1 - \exp\left(\frac{r}{b}\right)(1 - f_{\pi_0}(\theta_0)) \tag{24}$$

2. $-r < b \log \lambda < r$
   the bound is at least

$$\max_{\lambda} \left\{ \lambda f_{\pi_0}(\theta_0) - \lambda[1 - \frac{1}{2} \exp\left(-\frac{b \log \lambda + r}{2b}\right)] + \frac{1}{2} \exp\left(\frac{b \log \lambda - r}{2b}\right) \right\} \tag{25}$$

$$= \frac{1}{2} \exp\Big(-\log[2(1 - f_{\pi_0}(\theta_0)] - \frac{r}{b}\Big) \tag{26}$$

3. $b \log \lambda \leq -r$
   the bound is

$$\max_{\lambda \leq \exp\left(-\frac{r}{b}\right)} \lambda f_{\pi_0}(\theta_0) = f_{\pi_0}(\theta_0) \exp\left(-\frac{r}{b}\right) \tag{27}$$

Note that the lower bound is a concave function with respect to $\lambda$, then the prediction likelihood is:

$$\begin{cases} 1 - \exp\left(\frac{r}{b}\right)(1 - f_{\pi_0}(\theta_0)), & \text{when} \quad f_{\pi_0}(\theta_0) > 1 - \frac{1}{2}\exp\left(-\frac{r}{b}\right) \\ \frac{1}{2}\exp\left(-\log[2(1 - f_{\pi_0}(\theta_0)] - \frac{r}{b}\right), & \text{otherwise} \end{cases} \tag{28}$$

We have lower bound (28)$\geq \frac{1}{2}$ if and only if $r \leq -b \log[2(1 - f_{\pi_0}(\theta_0)]$, i.e., the analog DNN is robust within perturbation range $\mathcal{B} = \{\delta : \|\delta\|_1 \leq -b \log[2(1 - f_{\pi_0}(\theta_0)]\}$.

**Remark:** Thus, we have proved the robustness of analog DNN under the noise injection algorithmic framework. Note that this proof does not rely on specific assumptions of the analog DNN's architectures. The result can be directly extendable to different hardware implementations of analog DNNs.

**Supplementary Table 1**: Comparison with state-of-the-art reference work in terms of the type of noise injected, whether noise characteristics are optimized, whether device modifications are required and whether theoretical proof is provided.

| Papers | Injected noise considered | | | Optimality | Device prior | Theory guarantee |
|---|---|---|---|---|---|---|
| | Gaussian | Binomial | Laplace | | | |
| [7] | ✓ | X | X | X | Needed | X |
| [8] | ✓ | X | X | X | Needed | X |
| [9] | ✓ | X | X | X | Needed | X |
| [10] | ✓ | X | X | ✓(Bayes) | No Need | X |
| Ours | ✓ | ✓ | ✓ | ✓(Bayes) | No Need | ✓ |

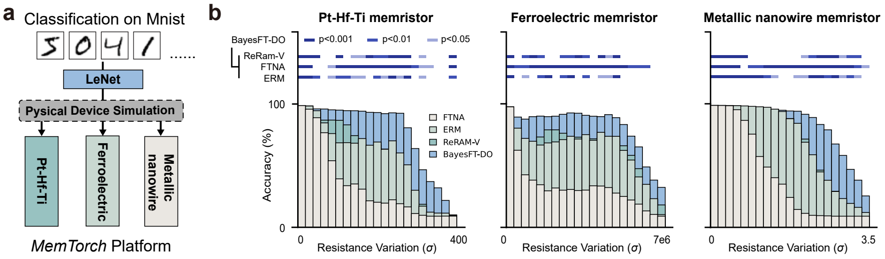


**Supplementary Figure S1: Mnist classification results on the MemTorch Platform. (a)Experimental settings on the MemTorch Platform. Pt-Hf-Ti, Ferroelectric, and Metallic nanowire were applied as three different types of pysical device simulation in the MemTorch.** (b)Experimental results on the *MemTorch* circuit simulation platform. The X-axis denotes the resistance variations and the Y-axis denotes the accuracies achieved by different training methods under resistance variations. The upper parts of the figures denote the results for statistical significance tests under different resistance variations. For example, for the Metallic nanowire memristor, the BayesFT-DO can achieve significantly better results than compared methods in the most regions of resistance variations.

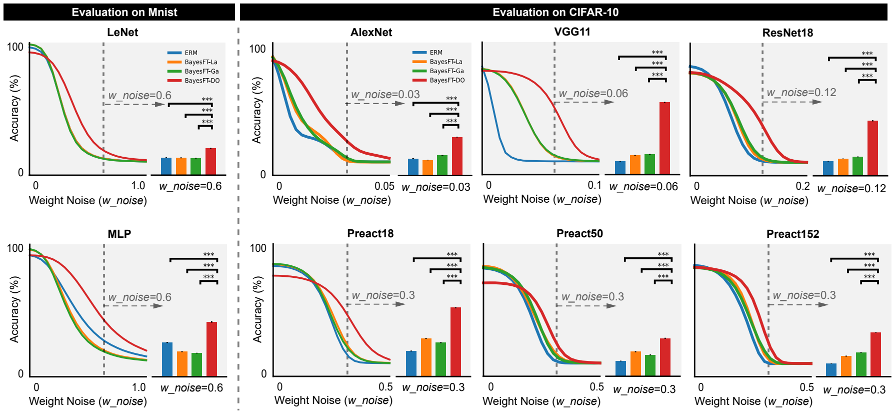


**Supplementary Figure S2: Experimental results on the *IBM-aihwkit* simulation platform.** Experiments were conducted on the Mnist and CIFAR-10 datasets with various neural network architectures. The curve charts compare the prediction accuracy of our methods (BayesFT-Ga, BayesFT-La, and BayesFT-Do) and the baseline methods (ERM) at different resistance variation ($\sigma$=0-1.5). The shaded areas are confidence intervals. The bar charts on the right with confidence intervals show the results of statistical tests (i.e. run the task for 20 times and compare the accuracy) at a specific weight noise level corresponded to the position of the vertical dotted line in the curve chart. The horizontal line above the bars indicates the statistical difference of the performance of our methods compared to the baseline methods (if the difference is significant, it will be marked by the $***$ symbol. Otherwise, the $p$-value will be displayed).

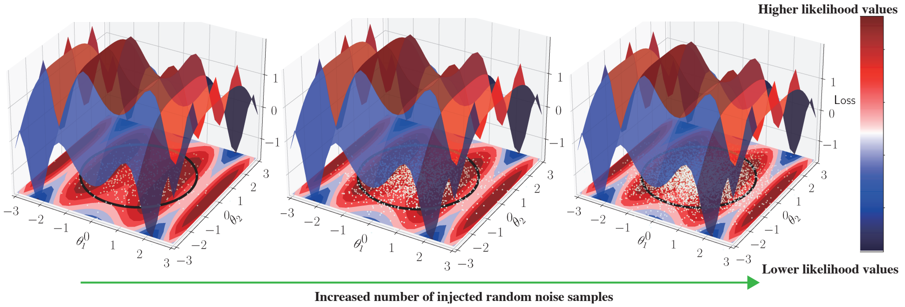


**Supplementary Figure S3**: **Intuitive explanations of theoretical proof.** Although the highly-complex likelihood function landscape with regard to parameters $\theta$ poses challenges for proving the robustness against analog noise, it is possible to investigate the likelihood landscape within a radius around the original parameters via random sampling of the landscape. With the increase of the injected random noise samples (white points in the figure), we can deduce the lowest possible likelihood value based on the noise spectrum and its sampled values within a space around the original parameter (the black circle in the figure).

# Supplementary Reference


[1] Corey Lammie, Wei Xiang, Bernabé Linares-Barranco, and Mostafa Rahimi Azghadi. Memtorch: An open-source simulation framework for memristive deep learning systems. *Neurocomputing*, 485:124–133, 2022.

[2] Shahar Kvatinsky, Misbah Ramadan, Eby G Friedman, and Avinoam Kolodny. Vteam: A general model for voltage-controlled memristors. *IEEE Transactions on Circuits and Systems II: Express Briefs*, 62(8):786–790, 2015.

[3] Resistive switching in $HfO_2$ probed by a metal–insulator–semiconductor bipolar transistor. *IEEE electron device letters*, 33(1):11–13, 2011.

[4] André Chanthbouala, Vincent Garcia, Ryan O Cherifi, Karim Bouzehouane, Stéphane Fusil, Xavier Moya, Stéphane Xavier, Hiroyuki Yamada, Cyrile Deranlot, Neil D Mathur, et al. A ferroelectric memristor. *Nature Materials*, 11(10):860–864, 2012.

[5] SL Johnson, A Sundararajan, DP Hunley, and DR Strachan. Memristive switching of single-component metallic nanowires. *Nanotechnology*, 21(12):125204, 2010.

[6] Malte J Rasch, Diego Moreda, Tayfun Gokmen, Manuel Le Gallo, Fabio Carta, Cindy Goldberg, Kaoutar El Maghraoui, Abu Sebastian, and Vijay Narayanan. A flexible and fast pytorch toolkit for simulating training and inference on analog crossbar arrays. In *2021 IEEE 3rd international conference on artificial intelligence circuits and systems*, pages 1–4, 2021.

[7] Weier Wan, Rajkumar Kubendran, Clemens Schaefer, Sukru Burc Eryilmaz, Wenqiang Zhang, Dabin Wu, Stephen Deiss, Priyanka Raina, He Qian, Bin Gao, et al. A compute-in-memory chip based on resistive random-access memory. *Nature*, 608(7923):504–512, 2022.

[8] Weier Wan, Rajkumar Kubendran, Clemens Schaefer, S Burc Eryilmaz, Wenqiang Zhang, Dabin Wu, Stephen Deiss, Priyanka Raina, He Qian, Bin Gao, et al. Edge ai without compromise: Efficient, versatile and accurate neurocomputing in resistive random-access memory. *arXiv preprint arXiv:2108.07879*, 2021.

[9] Christopher H Bennett, T Patrick Xiao, Ryan Dellana, Ben Feinberg, Sapan Agarwal, Matthew J Marinella, Vineet Agrawal, Venkatraman Prabhakar, Krishnaswamy Ramkumar, Long Hinh, et al. Device-aware inference operations in sonos nonvolatile memory arrays. In *2020 IEEE International Reliability Physics Symposium*, pages 1–6, 2020.

[10] Di Gaol, Grace Li Zhang, Xunzhao Yin, Bing Li, Ulf Schlichtmann, and Cheng Zhuo. Reliable memristor-based neuromorphic design using variation-and defect-aware training. In *2021 IEEE/ACM International Conference On Computer Aided Design*, pages 1–9, 2021.
